# EtoGel for Intra-Articular Drug Delivery: A New Challenge for Joint Diseases Treatment

**DOI:** 10.3390/jfb12020034

**Published:** 2021-05-16

**Authors:** Maria Chiara Cristiano, Antonia Mancuso, Elena Giuliano, Donato Cosco, Donatella Paolino, Massimo Fresta

**Affiliations:** 1 Department of Experimental and Clinical Medicine, Campus Universitario—Germaneto, “Magna Græcia” University of Catanzaro, Viale Europa, I-88100 Catanzaro, Italy; mchiara.cristiano@unicz.it; 2 Department of Health Science, Campus Universitario—Germaneto, “Magna Græcia” University of Catanzaro, Viale Europa, I-88100 Catanzaro, Italy; antonia.mancuso@unicz.it (A.M.); elena.giuliano@unicz.it (E.G.); donatocosco@unicz.it (D.C.)

**Keywords:** ethosomes^®^, intra-articular administration, poloxamer gel, rheology, arthritis

## Abstract

Ethosomes^®^ have been proposed as potential intra-articular drug delivery devices, in order to obtain a longer residence time of the delivered drug in the knee joint. To this aim, the conventional composition and preparation method were modified. Ethosomes^®^ were prepared by using a low ethanol concentration and carrying out a vesicle extrusion during the preparation. The modified composition did not affect the deformability of ethosomes^®^, a typical feature of this colloidal vesicular topical carrier. The maintenance of sufficient deformability bodes well for an effective ethosome^®^ application in the treatment of joint pathologies because they should be able to go beyond the pores of the dense collagen II network. The investigated ethosomes^®^ were inserted in a three-dimensional network of thermo-sensitive poloxamer gel (EtoGel) to improve the residence time in the joint. Rheological experiments evidenced that EtoGel could allow an easy intra-articular injection at room temperature and hence transform itself in gel form at body temperature into the joint. Furthermore, EtoGel seemed to be able to support the knee joint during walking and running. In vitro studies demonstrated that the amount of used ethanol did not affect the viability of human chondrocytes and nanocarriers were also able to suitably interact with cells.

## 1. Introduction

Rheumatic diseases are often degenerative joint diseases, e.g., osteoarthritis (OA) and rheumatoid arthritis (RA), and they are actually treated with oral, parenteral and intra-articular therapies [1]. The oral administration route has the best patients’ compliance but the administered dose is limited by the hepatic first pass effect and some drugs can cause gastrointestinal side effects [2]. Instead, intra-articular drug administration seems to be the most effective approach to treat joint diseases, because the local drug delivery is able to minimize side effects of drugs which are often administered through the systemic route [3]. On the other hand, the current intra-articular treatments require frequent and repeated injections due to a rapid degradation of injected drugs caused by the micro-environment of the joint and as a consequence they entail a high financial burden and reduction in patients’ compliance and quality of life [4].

Nonsteroidal anti-inflammatory drugs (NSAIDs) are frequently used for the therapeutic treatment of rheumatic joint diseases because they can reduce short-term pain in osteoarthritis of the knee [5]. Unfortunately, this category of drugs cannot be used for long-term therapy due to serious adverse effects associated with their oral administration [6,7]. NSAIDs could be intra-articularly administered to avoid gastrointestinal effects but they are characterized by a short synovial half-life and frequent injections are required to maintain therapeutic intra-articular levels [2,8]. In these cases, the smart approach may be the use of drug delivery systems acting as depot systems directly administered within the joint. In this way, drug retention time in joints can be prolonged, drug clearance and the number of administrations reduced and, consequently, the patients’ compliance can be increased [9].

Various drug delivery systems were proposed for intra-articular injections, i.e., liposomes [10,11,12], nanoparticles [13,14,15] and microparticles [16,17,18]. The intra-articular administration of drug-loaded delivery devices is also advantageous due to carrier ability to entrap drugs with different physicochemical features [19,20], to control the drug release and to protect their content against the joint micro-environment.

The physico-chemical features of drug delivery systems are key parameters to ensure their effectiveness following intra-articular administration. Namely, the shape of vesicles is important for controlling and minimizing the immune response; in fact, round-shaped systems are preferred for intra-articular administration compared with irregularly shaped ones, thus avoiding inflammatory reactions [1,21]. In addition to the shape, particle size must also be taken into account; in fact, it was demonstrated that an average particle size ranging from 160 nm to 750 nm was able to ensure a 2.6-fold increase in the retention time of dexamethasone-loaded liposomes following their joint injections [22]. Nanoparticles with a mean size of 38 nm were proposed for intra-tissue drug release in articular cartilage [23,24], thus being able to increase up to 72-fold the targeting of the extracellular compartment of articular cartilage with respect to larger particles. This finding was due to the pore size of the dense collagen II network that is approximately 60 nm [24]. Another important feature to consider during the design and preparation of nanosystems was the surface charge. The main drug delivery systems were characterized by a net negative or positive surface charge, that guarantees a strong repulsion between the nanosystems, thus avoiding instability phenomena such as aggregation and sedimentation [25,26]. Moreover, some research groups have demonstrated that negatively charged nanocarriers were characterized by better tissue compatibility, better cellular uptake and being related to a lower induction of inflammation than positively charged systems in intra-articular delivery [27,28].

Starting from this evidence, we focused our attention on the investigation of ethosomes^®^, elastic and deformable vesicles, which should be able to satisfy all the requirements to be used as a valid delivery device for intra-articular drug administration in the therapeutic treatment of joint disease. Ethosomes^®^ were described for the first time by Touitou et al. [29] as a new generation of liposomes characterized by a great amount of ethanol in their composition and have been used for topical applications such as skin drug delivery systems. Despite the high amount of ethanol in their composition, ethosomes^®^ have been shown to be safe in humans following their topical skin administration [30]. Ethosomes^®^ are characterized by the following features: suitable encapsulation efficiency for both hydrophobic and hydrophilic drugs [31], average particle size ranging from 100 nm to 300 nm as a function of ethanol and lipid amounts [32,33], closed spherical shape [34], great vesicular elasticity and deformability [35], which are absolutely useful for a colloidal carrier to be proposed for intra-articular drug administration.

These considerations prompted us to study ethosomes^®^ for a different use from the topical one initially proposed. Therefore, the aim of this work was the investigation of ethosomes^®^ as an innovative intra-articular drug delivery system (no study has been reported to the best of our knowledge), in order to obtain vesicular colloidal carriers characterized by suitable average particle size according to previously reported indications and deformability to easily cross the pores of dense collagen II network [22]. Moreover, ethosomes^®^ were embedded in a poloxamer P407 hydrogel to obtain many benefits. The embedding of ethosomes^®^ into poloxamer gel could be a promising strategy for obtaining an increased residence time of ethosomes^®^ in the joint environmental; thanks to its thermoreversible behavior, it could permit an easy intra-articular administration and reduce the rapid efflux of nanosystems which could occur if they were administered as suspension; moreover, the gel could protect the nanosystems from an in vivo rapid degradation. Another important advantage of the use of a gel–ethosome^®^ combination could be the possibility to simultaneously deliver drugs characterized by different hydrofily/lipophilicity, such as hydrophilic, lipophilic and amphiphilic active ingredients, obtaining the therapeutic effect of different drugs with a single intra-articular injection.

## 2. Materials and Methods

### 2.1. Materials

Phospholipon 90G^®^ (PL-90G) was kindly provided by Natterman Phospholipid GMBH (Köln, Germany), and contained 93 ± 3% phosphatidylcholine. Absolute ethanol (Ph. Eur. analysis reagent) and poloxamer 407 (P407) were purchased from Sigma-Aldrich (Schnelldorf, Germany). T/C-28a2 and C-28/I2 cells, immortalized chondrocyte cell lines, were obtained from the Research Foundation for the care of cancer “Tommaso Campanella” University Campus of Germaneto–Catanzaro, I-88100, Italy. Dulbecco-modified Eagle medium (D-MEM) culture, fetal bovine serum, penicillin (100 UI/mL)–streptomycin (100 μg/mL) solution (1% *v*/*v*), and Trypsin/EDTA (1×) solution were obtained from GIBCO (Invitrogen Corporation, Giuliano Milanese, Milan, Italy). 3-[4,5-dimethylthiazol-2-yl]-3,5-diphenyltetrazolium bromide (MTT) dye test (TLC purity ≥ 97.5%), phosphate buffer (PBS) solution, and sodium dimethyl sulfoxide (DMSO), were purchased from Sigma-Aldrich (Milan, Italy).

[^3^H]cholesteryl hexadecyl ether ([^3^H]CHE, 40 Ci/mmoL) was obtained from Perkin Elmer-Italia (Monza, Italy). Double distilled pyrogen-free water was used throughout the experimental investigations. All other materials and solvents used in this study are of analytical grade (Carlo Erba, Milan, Italy).

### 2.2. Methods

#### 2.2.1. Preparation of Ethosome^®^ Suspensions

Ethosomes^®^ with two different compositions were prepared by using 15% or 40% (*w*/*w*) ethanol, 1% (*w*/*w*) PL-90G and water up to 100% (*w*/*w*) (Table 1). Ethosomes^®^ with the greatest ethanol content (formulation A) were prepared as previously reported [33]. Briefly, PL-90G was placed in a Pyrex^®^ vial, solubilized in ethanol (Vortex, VELP Scientifica S.r.l., Monza and Brianza, Italy) and then the vial was hermetically sealed using a perforated cap to which an injection system was connected, thus reducing the solvent evaporation as much as possible. Double-distilled water was suitably added (up to 100% *w*/*w*) drop by drop at a rate of 200 μL/min until a milky suspension was obtained. The ethosomes^®^ were then finely homogenized (15,000 rpm × 1 min) using an Ultra-Turrax T25 equipped with the S25N-8G probe (IKA-WERKE). The final milky suspension of ethosomes^®^ was left at room temperature for 30 min under continuous stirring (Orbital Shaker KS 130 Control, IKA-WERKE).

Ethosomes^®^ with a low concentration of ethanol (15% *w*/*w*) (formulation B) were prepared by carrying out the first stage as in the procedure described above, and then an extrusion process to ensure the colloidal carrier stability. A Lipex Extruder™ (Vancouver, BC, Canada) and polycarbonate membranes with a 400 nm cut off (Costar, Corning Incorporated, New York, NY, USA) were used and ten extrusion cycles through two stacked membranes were carried out for each preparation.

For in vitro studies, ethosomes^®^ were sterilized following the sterilizing filtration method and by using 0.22 µm sterile filter.

#### 2.2.2. Ethosomes^®^ Characterization and Stability Evaluation

Mean size, polydispersity index (PdI) and zeta potential were evaluated by photon correlation spectroscopy (ZetaSizer Nano ZS, Malven Instruments Ltd., Worcestorshire, UK) [36]. The experiments were carried out using a 4.5 mW laser diode operating at 670 nm as a light source. Ethosome^®^ samples were suitably diluted with filtered water–ethanol mixtures at the same ratio used for ethosome^®^ preparation (Sartorius membrane filters 0.22 μm), thus avoiding the multiscattering phenomena, and then placed in quartz cuvettes. Mean size and size distribution data of various ethosome^®^ formulations are the mean of five different batches ± standard deviation.

The stability of ethosomes^®^ was evaluated using a Turbiscan Lab^®^ Expert instrument [37,38], equipped with a Turbiscan Lab Cooler. Briefly, samples were placed into a cylindrical glass tube and measurements were carried out for 3 h. The photon which was transmitted (T) and backscattered (BS) through the whole height (6 mm) of the sample was recorded. Analysis was carried out at 24 ± 1 °C and TurbiSoft software (Formulaction, L’Union, France) was used for data collection and evaluation of the kinetic stability of ethosome^®^ formulations.

#### 2.2.3. Deformability Index

The deformability index (DI) of ethosomes^®^ was determined as described previously [39]. Vesicles were extruded at a constant pressure (5 bar) through polycarbonate filters with a pore mean size of one third with respect to the initial mean size of ethosomes^®^. The DI was calculated according to the following equation:

(1) $$DI=J\left(\frac{d0}{p}\right)\left(\frac{d0}{d0-d1}\right)$$

where *J* is the fraction of suspension recovered after extrusion; *d*0 and *d*1 are vesicles’ mean size before and after extrusion, respectively; *p* is the membrane pore size. Data were expressed as the average of five different experiments ± standard deviation. Liposomes were used as a negative control of deformability [40].

#### 2.2.4. Cell Culture and Experimental In Vitro Methods

The human chondrocyte cell lines T/C-28a2 and C-28/I2 were incubated in plastic culture dishes (100 mm × 20 mm) at 37 °C and 5% CO2 in D-MEM medium with 10% FBS, penicillin (100 UI/mL), streptomycin (100 μg/mL) and amphotericin B (250 μg/mL) [41]. Every 48 h, fresh medium was substituted. When the cells reached a ~80% confluence, 2 mL of trypsin were used to detach cells, which were subsequently transferred into a centrifuge tube using 4 mL of fresh culture medium. The tube was centrifuged at 1000 rpm at room temperature for 5 min with an Eppendorf Centrifuge 5810. The pellet was resuspended in a suitable volume of culture medium and then seeded in culture dishes before in vitro investigation.

##### Cell Viability Assay

MTT test was carried out on T/C-28a2 and C-28/I2 cell lines to evaluate the in vitro cytotoxicity of ethosomes^®^. Cells were seeded in 96-well plates in 200 µL of medium at a density of 7000 cells/well. After 24 h, a fresh normal medium was supplemented with increasing concentrations (µg/mL) of ethosomes^®^ or an amount of ethanol equal to that used for their preparation. Every plate had 8 wells with untreated cells as the control. Cells were incubated for 24, 48 and 72 h. At the end of each treatment time, 10 µL of MTT (5 mg/mL dissolved in PBS solution) were added to each well and incubated for 3 h. Supernatants were removed and DMSO/ethanol solution (1:1 *v*/*v*) (200 μL) was added to dissolve the colored formazan crystals. The absorbance of the various samples was analyzed using an ELISA microplate reader (BIO RAD, xMark™ Microplate Absorbance Spectrophotometer, Hercules, California, USA) at λabs 570 nm and λem 670 nm. The percentage of cell viability was calculated according to the following Equation (2):

(2) $$\mathrm{cell}\;\mathrm{viability}\;\left(\%\right)=\frac{\mathrm{Abs}\;T}{\mathrm{Abs}\;C}\times 100$$

where *AbsT* is the absorbance of treated cells and *AbsC* is the absorbance of untreated cells. The results were the average of five different experiments ± standard deviation [33].

##### Ethosomes^®^–Chondrocytes Interaction

The interaction between low ethanol concentration ethosomes^®^ and T/C-28a2 or C-28/I2 cells as a function of time (1, 6 and 24 h) was evaluated as previously described by Cosco et al. [42]. Briefly, [^3^H]-CHE-radiolabeled (0.003% *w*/*w* corresponding to 3 nmol of [^3^H]-CHE) ethosomes^®^ were used. Radiolabeled ethosomes^®^ were purified from unintegrated [^3^H]-CHE molecules by means of centrifugation (80,000× *g* for 30 min at 4 °C). Cells were plated in six-well culture dishes (5 × 10^5^ cells/mL) and subsequently treated with 100 µL of [^3^H]-CHE-radiolabeled ethosomes^®^ in 2 mL of fresh medium for each well. After incubation times, cells were gently scraped and collected into centrifuge tubes, centrifuged (1200 rpm at room temperature for 10 min) to eliminate culture medium and uninternalized ethosomes^®^, washed twice with PBS, transferred into polypropylene liquid scintillation vials (20 mL size) (Sigma-Aldrich Chemie, GmbH, Steinheim, Germany), and dissolved in 2 mL of a quaternary ammonium hydroxide solution (BTS-450, Beckman Instruments Inc., Fullerton, CA, USA) under continuous shaking for 1 h at 60 °C using an incubator shaker (Innova 4300, New Brunswick Scientific, Edison, NJ, USA). After this incubation period, samples were diluted with 7 mL of liquid scintillation cocktail (Ready Organic, Beckman Coulter Inc., Fullerton, CA, USA) and then analyzed using a Wallac Win Spectral 1414 liquid scintillation counter (PerkinElmer Life and Analytical Sciences Inc., Waltham, MA, USA). A 1414 Win Spectral Wallac LCS software program was used for data analysis. Data are the average of five different experiments ± standard deviation.

#### 2.2.5. EtoGel Preparation

Thermosensitive hydrogels embedding ethosomes^®^ were prepared according to the cold method [43]. Briefly, an amount of P407 (15% *w*/*w*) was solubilized with double distilled water under continuous magnetic stirring at 4 °C. This hydrocolloid dispersion was stored at 4 °C until a clear hydrocolloid solution was obtained. After 24 h, ethosomes^®^ were added (2% *w*/*w*) under magnetic stirring [44]. Samples were then stored at 4 °C for 24 h before proceeding with the subsequent analyses.

#### 2.2.6. Microrheological Characterization of EtoGel

Rheolaser Master™ was used for the microrheological characterization of EtoGel. Rheolaser Master™ analysis is based on multi-speckle diffusing wave spectroscopy (MSDWS), which corresponds to dynamic light scattering [36]. Rheolaser Master™ analysis allows the sample characterization at rest, without any mechanical stress and without modifying the sample structure. Poloxamer 407 gel or EtoGel were placed in a 4 mL glass vial and in an appropriate Rheolaser Master™ adapter. The vial and adapter were placed in the sample chamber of the Rheolaser Master™ instrument where a constant laser beam is emitted that monitors the Brownian motion of dispersed particles. When the particles were hit by the light beam, the light was scattered and a speckle image was created. On the resulting graph, the Brownian motion of particles was reported in terms of “mean squared displacement” (MSD) versus time. Finally, Rheosoft Master 1.4.0 software processed the obtained data, which were expressed as MSD vs. decorrelation time curves and elasticity index (EI).

#### 2.2.7. Dynamic Rheological Characterization of EtoGel

A Kinexus Pro+ rotational rheometer (Malvern Instruments Ltd., Worcestershire, UK), equipped with cone–plate geometries (diameter 40 mm, angle 2°), was used for different rheological tests. A fixed gap between the geometries was pre-set to 1 mm and the excess sample was removed. Rheological data were processed by rSpace software. Compressed air flow (2 bar), pre-filtered through fine and superfine Clearpoint filters (Beko, Atlanta, GA, USA) was allowed to reach the pressure able to perform the analysis. The temperature during the analysis was measured and controlled by a high temperature cartridge (HTC) connected to the apparatus and equipped with a high accuracy PT100 sensor capable of stabilizing the temperature during the analysis. The samples were carefully and gently loaded onto the measuring plate of the rheometer and the measuring geometry was lowered at very slow speed, in order to prevent the alteration of the sample structure [45]. Before each analysis, the sample was kept at rest for 10 min.

##### “Syringe” Tests

The “syringe” tests were carried out according to a method developed in our lab to evaluate the EtoGel response to a simulated injection. The procedure consists of three steps: during the first step, the sample was subjected to a shear rate equal to 0.1 s^−1^ at room temperature, simulating a resting state of the formulation in the injection syringe; subsequently, the injection into the knee joint was simulated and shear rate and temperature were increased to 100 s^−1^ and 37 °C, respectively. This value of shear rate was instrumentally determined by the rSpace software, and it was calculated taking into consideration the diameter of a needle equal to 0.9 mm, normally used for intra-articular injection. The last step, corresponding to a simulated resting state of the sample into the knee joint, was characterized by a 0.1 s^−1^ shear rate and 37 °C.

##### Oscillatory Test with A Frequency Sweep

An oscillatory test with a frequency sweep ranging from 0.1 to 10 Hz was carried out at controlled strain (1%). The test was carried out at 37 °C to mimic body temperature and immediately after the syringe test. This range of frequency values was suitable to simulate movement of the joints during walking (0.5 Hz) and running (2.5 Hz) [46].

#### 2.2.8. Statistical Analysis

Statistical analysis of all experiments was performed by one-way ANOVA. A posteriori Bonferroni t-test was carried out to check the ANOVA test. A *p* value < 0.001 was considered statistically significant. Values are reported as the mean ± standard deviation.

## 3. Results

### 3.1. Physico-Chemical and Technological Characterization of Ethosomes^®^

In this study, a different administration route of ethosomes^®^ has been proposed. Ethosomes^®^ are usually designed as topical drug delivery systems typically characterized by high ethanol content (30–45% *w*/*w*) and a high deformability [33,47]. The ethanol content makes ethosomes^®^ unsuitable for applications other than skin. For this reason, we prepared ethosomes^®^ with a low ethanol concentration (15% *w*/*w*) (formulation B) for intra-articular administration and they were compared with an ethosome^®^ with an ethanol content of 40% (*w*/*w*) (formulation A). The various formulations were subjected to light scattering analysis to evaluate whether the reduced ethanol amount influences the mean size, polydispersity index and zeta potential. As shown in Table 1, mean sizes of formulation B were increased with respect to formulation A. This result was in agreement with Touitou’s study [29], which demonstrated that the ethosomes^®^ mean size increased when ethanol concentration decreased.

Owing to the preparation method and the extrusion phase, ethosomes^®^ with a suitable mean size for intra-articular administration were obtained. In fact, previous investigations demonstrated that a mean size greater than 160 nm elicited a marked increase in the intra-articular (knee joint) retention time of nanocarriers due to an efflux lag [22]. Furthermore, the extrusion step made it possible to obtain both a homogeneous ethosome^®^ suspension (PdI value lower than 0.3—Table 1) [48] and a fine control of the final mean size of systems in such a way to fulfill the therapeutic requirements.

The zeta potential analysis showed a net surface charge of the ethosome^®^ formulation B of −25.4 ± 0.4 mV, a zeta potential value sufficiently far from neutrality that ensures a suitable system stability [49]. In fact, a negative or positive surface charge elicits electrostatic repulsion, thus preventing agglomeration, flocculation and/or aggregation. These findings were in very good agreement with the stability analysis carried out using a Turbiscan Lab^®^ Expert (ALFATEST, Milan, Italy) [37]. The evaluation of colloidal stability as a function of time is a key step for the design of potential pharmaceutical nanocarriers.

**Figure 1:**
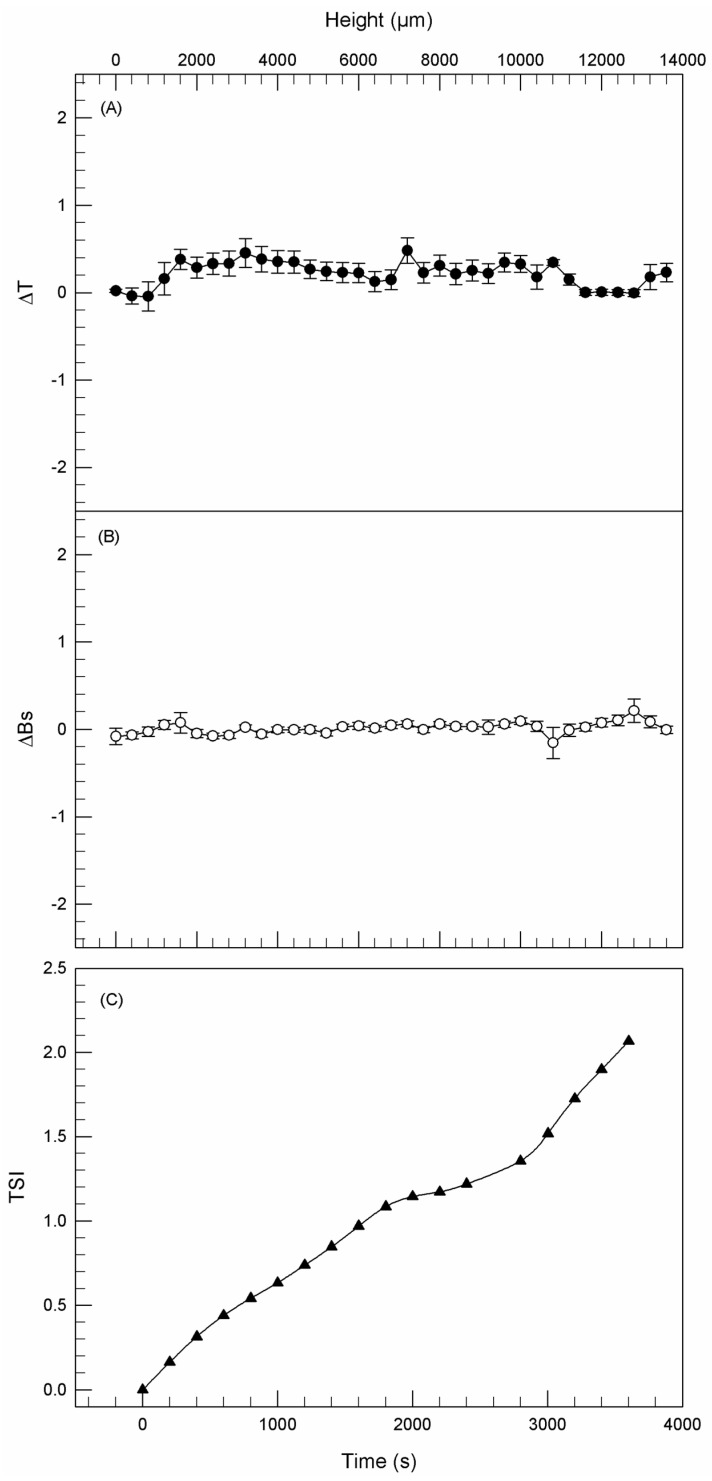
Delta transmission (ΔT—**A**), delta back scattering (ΔBS—**B**), and kinetic stability (TSI—**C**) profiles of formulation B. Panels report representative data from five independent experiments. Data are reported as a function of time (0–3 h) and sample height.

Figure 1A,B shows the backscattering (ΔBS) and transmission (ΔT) pathways of ethosome^®^ formulation B as a function of time (3 h), thus demonstrating that no significant modifications of both signals occurred during analysis. The variations of ΔT and ΔBS under 2 mm and above 8.5 mm of the sample height were not correlated with destabilization processes as previously demonstrated by Celia et al. [37], because these specific regions of the profile correspond to the bottom and the top of the cylindrical glass tube, respectively. Moreover, the kinetic stability profile fell within a narrow range of the Turbiscan Stability Index (TSI), thus demonstrating the absence of any colloidal destabilization phenomena (Figure 1C), as already observed for other ethosome^®^ formulations [33].

### 3.2. Deformability Evaluation

A suitable mean size is important for allowing the ethosome^®^ nanocarrier permeation within the synovial cavity, by considering that the dense collagen II network has pores with smaller diameters than the starting size of ethosomes^®^ [23]. Therefore, the particular features of ethosomes^®^, i.e., their deformability and elasticity, make them promising candidates for their innovative intra-articular application, thus being able to self-deform and cross through the small pores of the collagen II network. Due to the key role of the ethanol content for determination of the ethosome^®^ deformability feature [34], deformability evaluation of our ethosome^®^ formulation B was necessary to determine the maintenance of this characteristic even in the presence of low content of ethanol.

**Figure 2:**
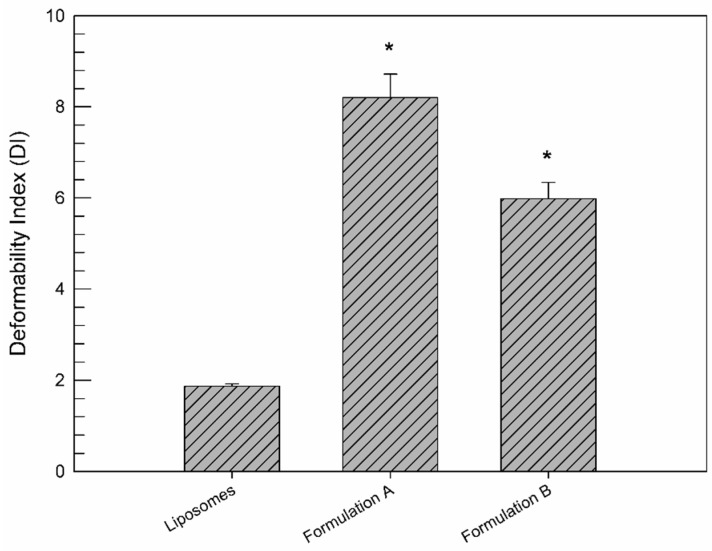
DI values of formulation B with respect to formulation A (as a positive control) and liposomes (as a negative control). Values represent the mean of five different experiments ± standard deviation. * *p* < 0.001 vs. liposomes.

As shown in Figure 2, the deformability features of ethosome^®^ formulation B were compared with those of ethosome^®^ formulation A and liposomes and were expressed as deformability index. Liposomes were used as a negative control, because they were not considered to be deformable vesicular nanocarriers [40]. The deformability of formulation B (DI = 5.98 ± 0.36) was reduced with respect to formulation A (DI = 8.20 ± 0.52), which is well recognized as a greatly deformable vesicular nanocarrier (positive control) [29]. This significant (*p* < 0.001) reduction, i.e., a 2.22 ΔDI, is due to the low ethanol amount used for formulation B preparation, which leads to a stiffening of the phospholipidic bilayer constituting the ethosomes^®^. On the contrary, the formulation B deformability is significantly greater (*p* < 0.001) than that of liposomes (DI = 1.87 ± 0.05), thus showing a 4.11 ΔDI. These findings clearly demonstrated the persistence of a suitable vesicle deformability of the ethosome^®^ formulation B, despite the reduced ethanol content, thus hypothesizing the ability of ethosome^®^ formulation B to cross through pores of the dense collagen II network. Furthermore, the low content of ethanol is surely a significant advantage in terms of intra-articular administration due to reduced chemically induced toxic effects.

### 3.3. MTT Test for In Vitro Cytotoxic Evaluation

By considering that the presence of ethanol and hence its potential toxicity may be a limiting factor for the intra-articular administration of ethosomes^®^, the cytotoxic profile of these innovative ethosomes^®^ was evaluated in vitro on human chondrocyte cell lines, i.e., T/C-28a2 and C-28/I2, by using the MTT test, which assayed cell viability as a function of both ethosome^®^ concentration and incubation time.

**Figure 3:**
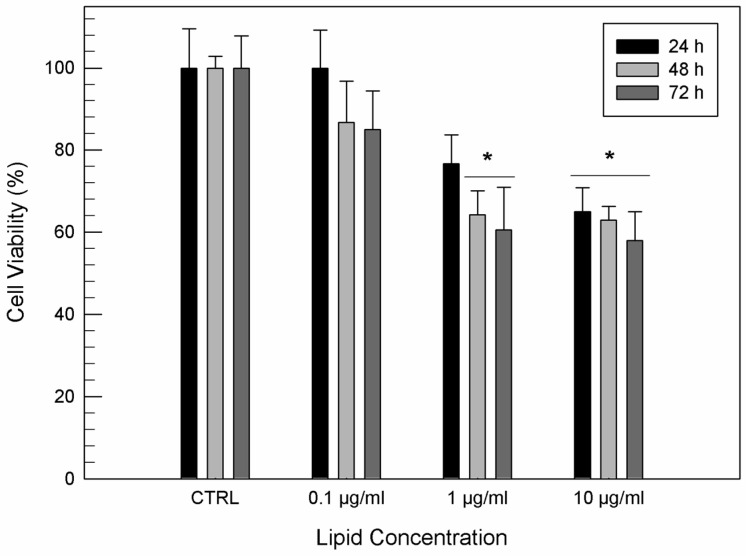
In vitro cytotoxicity of formulation B on T/C-28a2 cells as a function of the lipid concentration and incubation time. Results are the mean of five different experiments ± standard deviation. * *p* < 0.001 vs. control (untreated cells) at the same incubation time.

Formulation B showed no significant cytotoxic activity on T/C-28a2 human chondrocytes viability (Figure 3) with respect to the untreated cells for a lipid concentration of 0.1 µg/mL, independently of incubation times. A light cytotoxic effect of formulation B was observed at a lipid concentration of 1 µg/mL due to the presence of ethanol, after 48 h of exposure. When T/C-28a2 cells were treated with the highest lipid concentration of formulation, their viability was significantly reduced after 24 h of treatment.

To confirm that the resulting toxicity of formulation B was due to ethanol, another MTT study was carried out evaluating the effects of pure ethanol on human chondrocytes in comparison with formulation B. Indeed, formulation B at the highest investigated lipid concentration (10 µg/mL) and the same amount of ethanol used for its preparation were tested.

**Figure 4:**
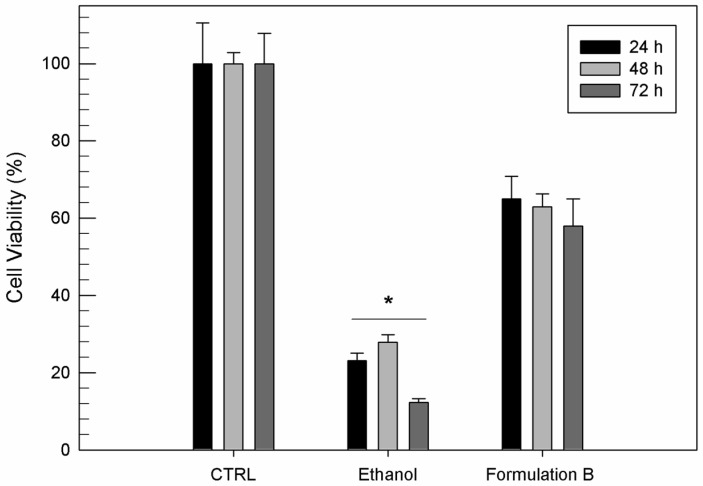
In vitro cytotoxicity of ethosome^®^ formulation B (10 µg/mL) compared to the same amount of free ethanol used for ethosome^®^ preparation on T/C-28a2 cells as a function of incubation time. Results are the mean of five different experiments ± standard deviation. * *p* < 0.001 vs. formulation B at the same incubation time.

As shown in Figure 4, ethosome^®^ formulation B was able to mask the toxic effect of free ethanol. The free ethanol induced a significant cell mortality (*p* < 0.001) already after 24 h of exposure, with a cell viability reduction of 80%, while the same amount of ethanol as a component of ethosomes^®^ elicited a minor reduction in cell viability after 24 h. After 72 h, the viability of cells treated with formulation B was three times greater than cells treated with the equal amount of free ethanol.

The in vitro cytotoxic activity evaluated on C-28/I2 cells gave similar results to those obtained with the T/C-28a2 cell line, in terms of both incubation time and lipid concentrations (data not shown).

### 3.4. Interaction between Ethosomes^®^ and Cell Lines

**Figure 5:**
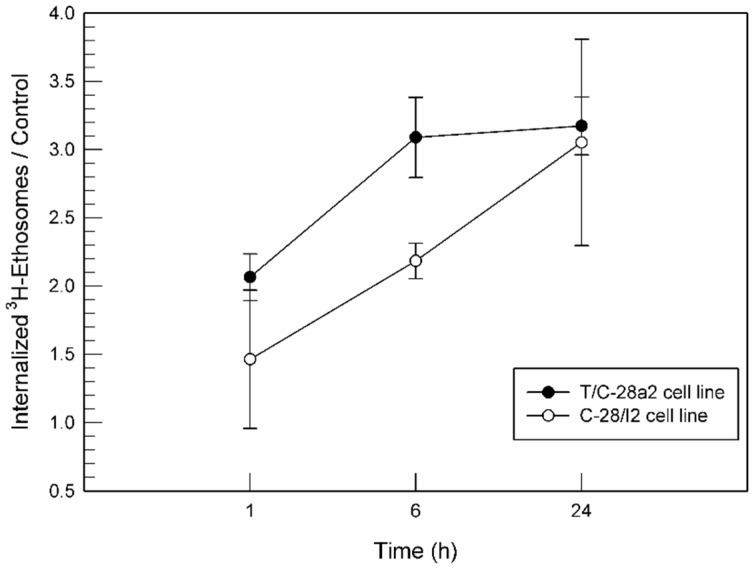
Interaction between [^3^H]CHE-radiolabeled ethosomes^®^ at a 0.1 µg/mL lipid concentration and the T/C-28a2 and C-28/I2 cell lines, normalized for untreated cells.

Ethosomes^®^ at low ethanol concentration may be considered effective drug delivery systems for intra-articular application whether they are able to interact with joint cells. To this aim, an investigation of the cell–nanosystems interaction using [^3^H]CHE-radiolabeled ethosomes^®^ was carried out. The obtained radiolabeled ethosomes^®^ were also characterized in terms of mean size, surface charge, and deformability. The results of this characterization were not statistically different from the physico-chemical characterization of empty ethosomes^®^, demonstrating that the content of [^3^H]cholesteryl hexadecyl ether used during preparation did not alter their features. According to the findings of in vitro MTT results, radiolabeled ethosomes^®^ at a lipid concentration of 0.1 µg/mL was used for cell interaction studies. As shown in Figure 5, the interaction seemed to be time- and cell line-dependent. In detail, a significant interaction was observed just after 1 h of treatment for both cell lines and it was also exposition-time dependent, i.e., the longer the incubation the greater the cellular interaction. As shown in Figure 5, T/C-28a2 and C-28/I2 had a different sensitivity to [^3^H]CHE-radiolabeled ethosomes^®^. T/C-28a2 cells showed a greater interaction rate towards ethosomes^®^ than C-28/I2 cells during the first 6 h. These findings suggested that T/C-28a2 were characterized by more permeable membrane structures and hence ethosomes^®^ were able to pass through the membrane more quickly than in the case of C-28/I2 cells. Anyhow, the interaction between ethosomes^®^ and cells was similar after 24 h of incubation.

### 3.5. Microrheological and Dynamic Rheological Characterization of EtoGel

In order to further prolong the residence time and joint retention of ethosomes^®^, they were embedded in poloxamer P407 gels, thus leading to a novel supramolecular drug delivery device, the so-called EtoGel. We chose to use poloxamer P407 instead of hyaluronic acid despite the fact that the latter is one of the most used components for visco-supplementation. Hyaluronic acid is widely used to ensure both a replacement of reduced endogenous hyaluronic acid and a visco-elasticity to the used formulations. Despite all the advantages of hyaluronic acid, a number of studies demonstrated several problems, which can have serious consequences for patients [50]. Some clinical studies have shown that after 24–48 h from the injection of exogenous hyaluronic acid, an inflammatory process can occur and pain, swelling and joint edema can be observed [51]. This inflammatory process is caused by the endogenous degradation of hyaluronic acid by synovial enzymes that produce pro-inflammatory mediators [52]. Another limitation of the intra-articular use of hyaluronic acid is the short duration of action, which is caused by the rapid efflux, and hence repeated administrations are required. All these drawbacks lead to a reduction in patient compliance and an increase in the therapy cost.

To obtain the same advantages generated by hyaluronic acid and, at the same time, to reduce the therapy cost and to increase patient compliance, we chose to use poloxamer P407, an amphiphilic linear polymer, which is recognized as a GRAS (generically recognized as safe) excipient [52], thus obtaining a thermo-reversible gel [53,54]. Poloxamer 407 gel showed a liquid-like behavior at low temperatures (i.e., room temperature) and a solid-like one at higher temperatures (i.e., at body temperature), depending on the poloxamer concentration [55]. The specific thermo-reversibility properties of poloxamer 407-based EtoGel should allow an easier intra-articular administration in the liquid state and, at the same time, a prolonged persistence in the gel.

#### 3.5.1. Microrheological Characterization

The microrheological characterization of a poloxamer gel and EtoGel was carried out using Rheolaser Master™ (Formulaction, Toulouse, France) [36].

The Rheolaser Master™ allowed the analysis of samples at rest without inducing any modification of the sample structure. In this way, the basic rheological properties of the sample can be detected and different samples can be compared, thus exploiting the Brownian motion of dispersed particles [56,57]. In particular, in the case of purely viscous samples, the embedded colloidal particles (ethosomes^®^ in this case) are characterized by free motion within a three-dimensional network and hence the resulting MSD curves grow linearly with decorrelation time. On the other hand, in the case of samples characterized by high fullness and viscoelastic features, the motion of the embedded colloidal particles is limited, the MSD shape is not linear and closer to the *x*-axis.

**Figure 6:**
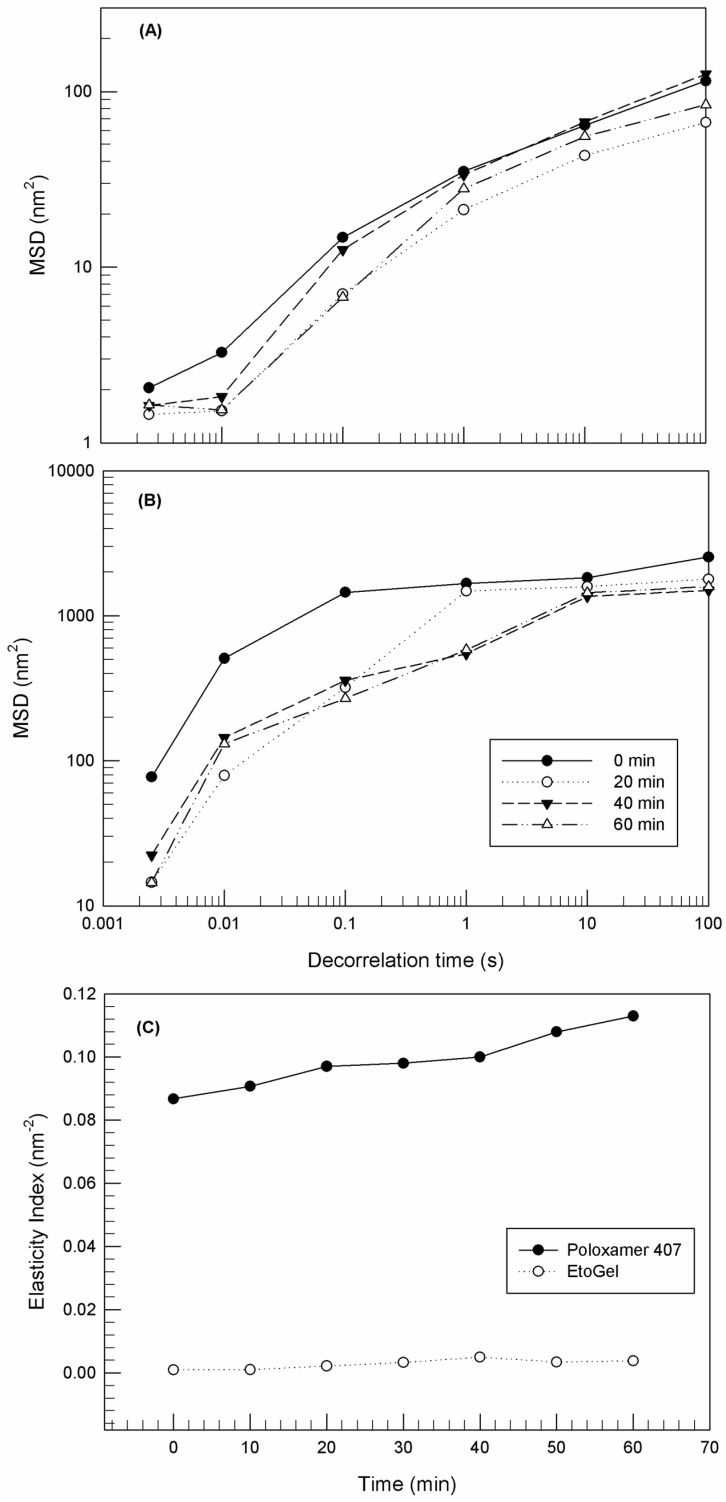
Mean square displacement (MSD) for Poloxamer gel (**A**) and EtoGel (**B**) as a function of decorrelation time, and elasticity index (EI) (**C**) of samples as a function of time. Experiments were carried out at 25 °C. Results were representative of five independent experiments.

Figure 6 shows the MSD curves at different aging times of poloxamer gel (panel A) and EtoGel (panel B). The MSD curves’ threshold of poloxamer gel showed lower values than EtoGel. Namely, the MSD curves of poloxamer gel reached maximum values of ~100 nm^2^, while the MSD curves of Etogel exceed values of ~1000 nm^2^. This marked difference between the MSD curves of the two samples is an index of different behavior at rest, i.e., EtoGel was less viscous than poloxamer gel and hence the particles inside the EtoGel move more freely [53]. This aspect is particularly evident in the elasticity index (EI) curves (Figure 6C). EI values are calculated from MSD curves and correspond to the inverse of particles’ movement velocity within the network. As shown in Figure 6C, the EI values of EtoGel were significantly lower than those of poloxamer gel, thus demonstrating the different movement freedom of the constituent particles of the two samples. Ethosomes^®^ seem to decrease the rigidity of the poloxamer network and hence particles seem to move more freely in EtoGel. Probably, this result was due to the presence of the ethanol as a constituent of EtoGel which could induce weaker bonding in the poloxamer network [36].

#### 3.5.2. Dynamic Rheological Studies

The rheological characterization of soft materials and the evaluation of the viscoelastic behavior of gels are fundamental aspects to evaluate during the design of new pharmaceutical products [58,59].

##### “Syringe Test”

**Figure 7:**
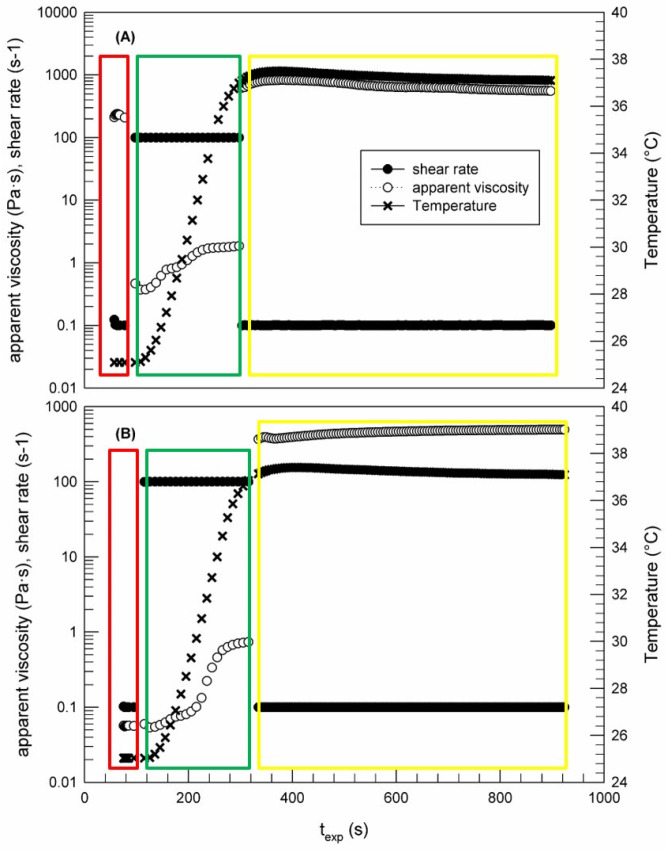
Syringe test carried out by Kinexus Rotational Rheometer to evaluate the viscosity curve of poloxamer gel (**A**) and EtoGel (**B**) as a function of temperature and the stage of intra-articular injection (shear rate). The result was a representative experiment of five independent experiments.

The “syringe test” is an innovative rheological analysis that we have just fine-tuned in our laboratory to evaluate the behavior of samples during a hypothetical intra-articular injection through a needle with a specific diameter, as shown in Figure 7, where rheological behaviors of poloxamer 407 gel (A) and EtoGel (B) were reported in terms of apparent viscosity. In particular, the analysis is based on three steps characterized by different shear rates and temperatures. In the first phase of the “syringe test” (red box), we simulated a resting phase of the poloxamer-based formulation in the syringe at room temperature, and a very low shear rate (0.1 s^−1^) was applied thus measuring its basal viscosity.

Samples of the Poloxamer 407 gel and EtoGel were characterized by a different starting viscosity, thus confirming the Rheolaser Master™ findings (Figure 6). Namely, the poloxamer gel viscosity at room temperature was 233.24 ± 3.67 Pa·s, while when ethosomes^®^ were embedded into the poloxamer gel the viscosity decreased to 0.056 ± 0.001 Pa·s, probably because ethosomes^®^ were able to negatively influence the three-dimensional network of the poloxamer gel.

In the second phase (green box), the shear rate was increased to 100 s^−1^, thus simulating an injection through a needle with a diameter of 0.9 mm and temperature was progressively increased to 37 °C. As soon as the shear rate is set to 100 s^−1^, the viscosity of poloxamer gel quickly decreased to a value of below of 1 Pa·s, thus demonstrating the dependence of poloxamer gel viscosity on shear rate. This finding is important in terms of clinical applicability because it demonstrated that poloxamer gel can be easily injected through a specific needle, despite the high starting viscosity. In fact, it would be difficult to think of a possible intra-articular administration if there is no reduction in the initial viscosity of a gel-like device following a certain stress application. EtoGel showed a different rheological behavior (Figure 7B). The sample viscosity was not influenced by the applied shear rate and it remained on the initial low values, without imposing resistance when passing through the needle.

Finally, the third phase of experiments (yellow box) was carried out to simulate the persistence of gel into the knee joint, thus setting experimental parameters to a minimum shear rate and body temperature. As shown in Figure 7B, when the shear rate was brought back to low values (0.1 s^−1^) at a temperature of 37 °C, EtoGel showed a greater viscosity value (383.45 ± 6.94 Pa·s) than that observed in the first experimental phase. This viscosity change was elicited by reaching the temperature of thesol–gel transition of the poloxamer gel, according to previous studies which demonstrated the existence of a 15% (*w*/*w*) poloxamer 407 solution in the gel state at a temperature of 37 °C [43]. A similar trend was observed for the ethosome^®^-free poloxamer gel, thus confirming that the embedded ethosomes^®^ did not influence the sol–gel transition of the poloxamer gel, despite the different starting viscosity at room temperature of the two samples.

These findings are very encouraging in terms of intra-articular administration, because they demonstrate that EtoGel viscosity at rest and room temperature can allow an easy injection into the joint, while the viscosity increase at body temperature will allow a longer time permanence of ethosomes^®^ and their payload within the joint.

##### Oscillatory Test with a Frequency Sweep

After demonstrating the injectability of EtoGel, it was essential to evaluate the rheological behavior of the post-injection samples, since it is assumed that you want to guarantee a life as normal as possible for patients who undergo intra-articular supplementation. The patient is supposed to carry out physical movements such as walking and running in the days following the injection. Some research groups have studied the rheological tests and frequency values necessary to simulate the walking and the running [46,60]. From these studies, the authors defined that 0.5 Hz and 2.5 Hz are the frequency values suitable to mimic the joint movements during walking and running, respectively.

**Figure 8:**
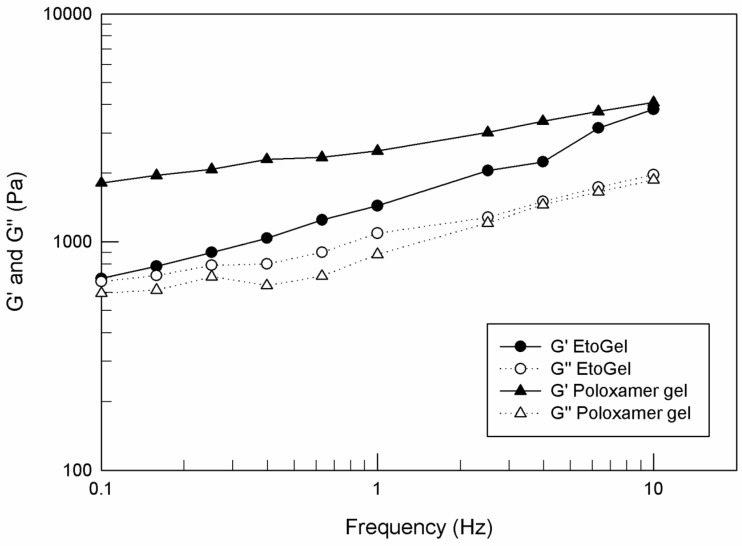
G′ and G″ curves (Pa) versus frequency (Hz), carried out by Kinexus Rotational Rheometer. The result was a representative experiment of five independent experiments.

For these purposes, we carried out another rheological analysis to characterize the viscous–elastic behavior of EtoGel following intra-articular injection at these frequency values, evaluating the elastic modulus G′ (Pa) and viscous modulus G″ (Pa) as a response to a frequency sweep. As shown in Figure 8, the G′ values are kept above the G″ values throughout the experimental frequency range for both analyzed samples. Moreover, no crossover frequency, at which the storage and loss moduli were equal, was observed. These results demonstrated the ability of EtoGel and poloxamer gel to maintain their gel characteristics without undergoing a gel–sol transformation following stress. Namely, in case of a gel transformation in a liquid-like form (values of the viscous modulus exceed the values of the elastic modulus) the injected formulation will leave the patient’s joint in such a time as not to allow a delivered drug to exert its’ in situ therapeutic action. This result demonstrated that the poloxamer gel, regardless of the ethosome^®^ inclusion, is able to support the joint during walking (0.5 Hz) and running (2.5 Hz) [46,60] and to maintain its gel characteristics.

## 4. Conclusions

Intra-articular administration of drugs is increasingly in demand because it overcomes all of the drawbacks that arise from conventional therapy using the oral administration of drugs. Unfortunately, direct drug administration in the joint has limitations mainly due to the rapid outflow from the cartilaginous environment. To this aim, our research group has designed a potential and new intra-articular system, represented by a combined system of modified ethosomes^®^ and a three-dimensional network. The first goal was the conception of deformable vesicular systems, the ethosomes^®^, but characterized by such a concentration of ethanol as not to alter the viability of the chondrocytes. Deformability is a fundamental requirement to ensure the cross through the pores of the dense collagen II network and the in-situ release of any drug. Moreover, the modified ethosomes^®^ were incorporated into a three-dimensional network of poloxamer P407, thus obtaining a thermo-sensitive EtoGel. This network can increase the residence time of nanosystems into joints and the rheological characterization demonstrated that EtoGel is suitable for easy intra-articular administration. Since the EtoGel has been shown to withstand the stresses deriving from a walk or a run, we can hypothesize a real applicability of EtoGel in the clinical setting for the resolution of pathologies affecting the joints, guaranteeing the patient in therapy an almost normal life.

## Figures and Tables

**Table 1:** Lipid composition and physico-chemical parameters of ethosomes^®.^

Formulation	Ethanol (% *w*/*w*)	PL90G ^a^ (% *w*/*w*)	H_2_O (% *w*/*w*)	Mean Size (nm)	PdI ^b^	Z-Potential (mV)
A	40	2	58	157 ± 1	0.122 ± 0.007	−48.0 ± 0.8
B	15	2	83	280 ± 1	0.116 ± 0.013	−25.4 ± 0.4

^a^ Phospholipon 90G. ^b^ Polydispersity index.

## Data Availability

The data presented in this study are available on request from the corresponding author. The data are not publicly available due to privacy.
